# The Heart Club: How Cyanotic Heart Disease Was Reframed

**DOI:** 10.3390/diseases5040022

**Published:** 2017-09-30

**Authors:** Tom Treasure

**Affiliations:** Clinical Operational Research Unit, University College London, Gower Street, London WC1E 6BT, UK; tom.treasure@gmail.com

In April 1948, the thoracic surgeon, Russell Brock, convened a meeting at Guy’s Hospital, London of “those concerned in the management of congenital disease of the heart” [1]. This marked the formal opening of a deliberate, carefully considered, and concerted effort to treat untreatable patients, and was to change the whole way of thinking about heart disease. The enterprise is described in my book “The Heart Club” which includes the minutes of its 47 consecutive meetings. Over a relatively short period of eight years, the Club contributed to the establishment of surgery for structural heart disease which has become one of the most effective of all surgical endeavours. The Guy’s group were not alone, but the solidarity of their approach, the rate of change in practice, and a uniquely insightful and methodical approach is chronicled in the transcript of the minutes. From its beginnings, the question became not whether, but how structural diseases of the heart could be fixed. If we consider the number of people who may gain benefit, the magnitude of the change, and the consistency with which it is now achieved, heart surgery ranks alongside joint replacement and cataract operations among the great surgical contributions of the 20th century. Brock’s team and others of a like mind changed the whole attitude to heart disease and, in the language of social and cultural history of medicine, they changed how heart disease was “framed” [2].

The evidence of the prior position is there in the medical texts, and in the teaching at the time that Brock convened his club. Guy’s senior physician, Sir John Conybeare, wrote in the 1946 edition of his book “cyanosis is the first sign and is so characteristic that “blue baby” and congenital heart disease are practically synonymous” [3]. Teaching the students, his colleague Maurice Campbell said “I do not intend to say much about the grossly cyanosed cases” because there was not much to be done apart from limiting their activity and giving their parents the gloomy but realistic prognosis: their child’s life would be short and fairly miserable [4]. This fatalism came with a holistic view of the heart. The mantra of James Mackenzie “A heart is what a heart can do” had become entrenched dogma. There had been some surgery for mitral stenosis in the 1920s which Mackenzie had decried. Bringing together all 10 known cases in a paper in 1929 the Boston surgeon Elliot Cutler titled his paper the “final report”. There had been glimmers of hope with a couple of patients surviving for a few years. These cases are pointed to in the “victor’s history‘’ of cardiac surgery but the cardiologists had a point. The 1920s experience had been largely disastrous. In the interests of their patients, cardiologists preached against any more attempts to operate on the heart and became more forthright in opposition up to the mid-1940s.

An important lever for change came with the systemic to pulmonary artery shunt operation. This was the brain child of Helen Taussig, worked out in the laboratory by Vivien Thomas, and brought into clinical practice by Alfred Blalock. Thanks to a bond forged in wartime medical services, an exchange programme was created between Guy’s and Johns Hopkins and in 1947 Blalock came to London and performed 10 shunt operations. The operation was tactically a knight’s move, brilliant but somewhat serendipitous. The surgery was not within, but adjacent to the heart. It altered the physiology, permitting more unsaturated blood to reach the heart, and got around the obstacle of the heart being a declared no-go area for surgeons. Blalock’s operation was adopted and the clinical work of the Club became established and legitimised. It was then possible for Brock to achieve his objective of getting to the root of the problem: intracardiac surgery was achieved. He soon wrote up his intracardiac operations to relieve the cyanosis of Fallot’s tetralogy, publishing alongside the Guy’s cases the results of seven operations performed while he was on a return visit to Johns Hopkins [5] (Figure 1).

As a result of the work of “The Cardiac Club” and others who came after, congenital heart disease was reframed as a surgical disease. Attention was focused once more on the exact anatomy so that surgical correction could be undertaken precisely and reproducibly. Achieving survival for formerly fatal malformations created another diagnostic frame with the coining of the phrase “grown up congenital heart disease”. “GUCH” is now more prosaically rebranded as adult congenital heart diseases. In the book of “The Heart Club”, three patients of that era who are still alive and well, tell their life stories. The book is a testimony to team work, resourcefulness and perseverance.

## Figures and Tables

**Figure 1:**
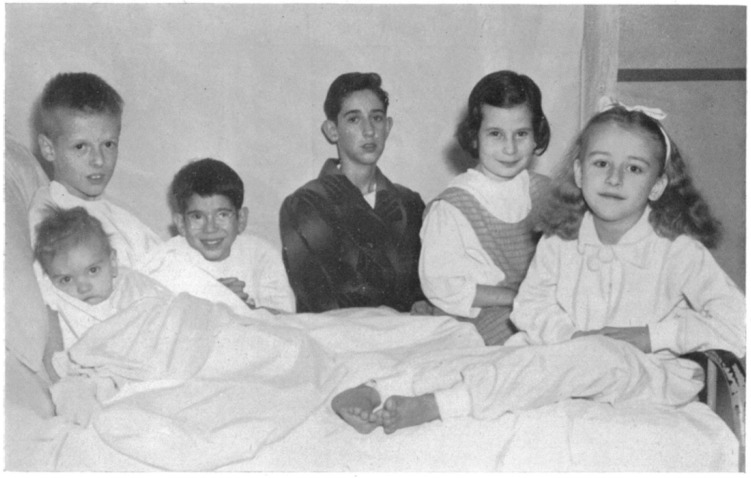
These are the six surviving, of seven children operated on by the Guy’s surgeon Russell Brock during a one month exchange visit to Baltimore in 1949 during which he introduced surgery for both pulmonary and mitral stenosis to his colleagues in Johns Hopkins.
